# 101. Patients with Carbapenem-resistant *Klebsiella aerogenes* and *Enterobacter cloacae* complex Colonization or Infection Have Different Baseline Characteristics but Similar Mortality and Clinical Outcomes

**DOI:** 10.1093/ofid/ofae631.038

**Published:** 2025-01-29

**Authors:** John P Franzone, Wanying Shao, Lauren Komarow, Jesse T Jacob, Michael J Satlin, Keith S Kaye, Blake M Hanson, Gregory D Weston, Bettina F Fries, Eric Cober, Sorabh Dhar, Jianping Jiang, Liang Chen, Carol Hill, Angelique E Boutzoukas, Tessa Andermann, David van Duin

**Affiliations:** University of North Carolina at Chapel Hill, Apex, NC; George Washington University, Rockville, Maryland; George Washington University, Rockville, Maryland; Emory University School of Medicine, Atlanta, GA; Weill Cornell Medicine, New York, NY; Rutgers Robert Wood Johnson Medical School, New Brunswick, NJ; The University of Texas Health Science Center, Houston, Texas; Montefiore Medical Center and Albert Einstein College of Medicine, Bronx, NY; Renaissance School of Medicine at Stony Brook University, Stony Brook, NY; Cleveland Clinic Foundation, Cleveland, OH; Wayne State University/Detroit Medical Center, John Dingell VAMC, Detroit, Michigan; Center for Discovery and Innovation, Hackensack Meridian Health, Nutley, New Jersey; University of Buffalo, Buffalo, New York; Duke Clinical Research Institute, Durham, North Carolina; Duke University School of Medicine, Durham, North Carolina; University of North Carolina at Chapel Hill, Apex, NC; University of North Carolina at Chapel Hill, Apex, NC

## Abstract

**Background:**

Recently, comparative phylogenetics dictated the formerly named *Enterobacter aerogenes* be separated from the genus *Enterobacter* and renamed *Klebsiella aerogenes* (KA); however, it remains unclear whether the genotypic differences responsible for the reclassification translate into clinical differences. We aimed to evaluate clinical characteristics and outcomes of patients colonized or infected with carbapenem-resistant (CR) *Enterobacter cloacae* complex (ECC) or CR KA.

**Figure:**
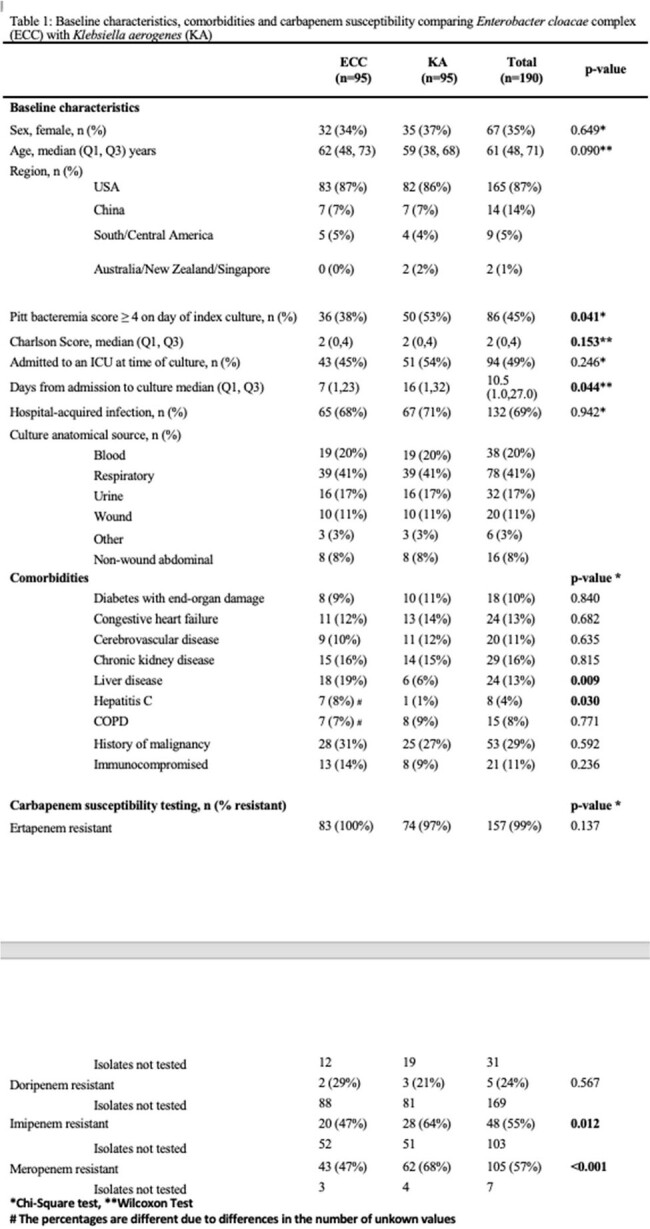

**Methods:**

We conducted a nested, case-control study of patients hospitalized from May 9^th^, 2016 until November 11^th^, 2019 and enrolled in The Consortium on Resistance Against Carbapenems in *Klebsiella* and other *Enterobacterales* II (CRACKLE-2). Cases were patients with an index culture with CR KA and controls were patients with an index culture positive for CR ECC, matched to each case by: 1) country, 2) anatomical source, 3) infection vs. colonization, and 4) availability/result of whole genome sequencing for the isolate. Where WGS was available the pan-genome Ortholog Clustering Tool was used to identify flexible genomic islands (FGI) associated with KA (n=65) versus ECC (n=66).

**Figure:**
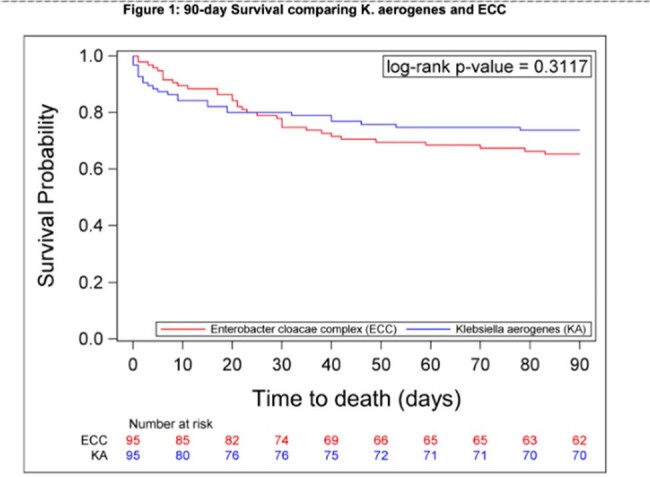

**Results:**

Cases with CR KA (n=95) were matched with 95 controls with CR ECC. 49% of cultures met infection criteria. When compared with control patients, cases with KA were less likely to have liver disease (6% vs. 19%,p=0.009) but more likely to have been admitted for longer at the time of culture (median [IQR] 16[1,32] vs. 7[1,23]), have a Pitt score ≥ 4 (53% vs. 38%, p=0.041), and to have been in an intensive care unit (ICU) at the time of culture (54% vs. 45%, p=0.246) (Table 1). 30-day mortality (21% vs. 25%, p=0.492), 90-day mortality (26% vs. 35% p=0.208), and 30-day DOOR outcomes (p=0.727) did not differ between cases and controls for all patients (Figure 1, 2). There were 94 unique FGI containing 216 putative virulence factors for KA versus 77 and132 for ECC.

**Figure:**
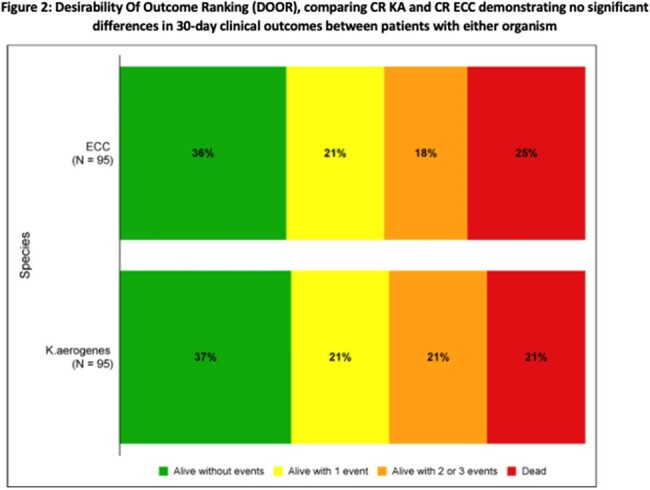

**Conclusion:**

Despite significant differences in baseline characteristics and in the putative virulence genes present, clinical outcomes are similar in patients with CR KA and CR ECC colonization and infection (alive without events 37% vs. 36%).

**Disclosures:**

**Michael J. Satlin, MD**, AbbVie: DSMB participant|bioMerieux: Grant/Research Support|Merck: Grant/Research Support|Selux Diagnostics: Grant/Research Support|SNIPRBiome: Grant/Research Support **Keith S. Kaye, MD, MPH**, Allecra: Advisor/Consultant|CARB-X: Advisor/Consultant|GSK: Advisor/Consultant|Merck: Advisor/Consultant|Shionogi: Advisor/Consultant|Spero: Advisor/Consultant **Carol Hill, PhD**, Glaxo SmithKline: Retirement Health, Cash Balance Plan|Glaxo SmithKline: Stocks/Bonds (Public Company) **David van Duin, MD, PhD**, Merck: Advisor/Consultant|Merck: Grant/Research Support|Pfizer: Advisor/Consultant|Qpex: Advisor/Consultant|Roche: Advisor/Consultant|Shionogi: Advisor/Consultant|Shionogi: Grant/Research Support

